# Mg²⁺-modified corncob biochar and natural zeolite significantly enhanced simultaneous N and P recovery from livestock wastewater

**DOI:** 10.1371/journal.pone.0331575

**Published:** 2025-08-29

**Authors:** Yu Jin, Haifeng Wu, Shuai Huo, Siyu Chen, Lili Shen, Hongjie Di, Lianfeng Du, Peng Li

**Affiliations:** 1 College of Resources and Environment, Shanxi Agricultural University, Jinzhong City, Shanxi Province, China; 2 Institute of Plant Nutrition, Resources and Environment, Beijing Academy of Agriculture and Forestry Sciences, Beijing, China; 3 China-New Zealand Joint Laboratory of Water Environment Research, Beijing Academy of Agriculture and Forestry Sciences, Beijing, China; 4 Centre for Soil and Environmental Resource, Lincoln University, Lincoln, New Zealand; National Chung Cheng University, Taiwan & Australian Center for Sustainable Development Research and Innovation (ACSDRI), AUSTRALIA

## Abstract

Livestock and poultry farming effluents (LPFE) have become a significant source of pollution in the agricultural water environment, with nitrogen (N) and phosphorus (P) being the major pollutants. The use of inexpensive Mg2^+^ -modified materials as crystallization carriers can enhance the struvite crystallization and precipitation reaction, enabling efficient recovery of N and P nutrients from LPFE. In this study, Mg2^+^ -modified corncob biochar (CCBC-Mg) and Mg2^+^ -modified natural zeolite (NZ-Mg) were assessed for their simultaneous recovery of N and P from LPFE. BET, SEM-EDS, FTIR, XRD and adsorption isotherm and adsorption kinetics modelling were used to study the N and P adsorption properties and mechanisms. The results showed that Mg2^+^ modification significantly enhanced the simultaneous ammonia nitrogen (AN) and total phosphorus (TP) recovery of the two materials. After modification, corncob biochar (CCBC-Mg) showed the best P recovery performance, while natural zeolite (NZ-Mg) showed the best AN recovery, with adsorption quantities reaching 116.99 mg/g and 47.50 mg/g, respectively. The findings demonstrated that the adsorption processes for both AN and phosphate across all materials adhered to the pseudo-second-order kinetic model and Langmuir isotherm, collectively indicating that monolayer adsorption governed by chemical interactions was the predominant mechanism. CCBC and NZ mainly adsorbed AN and TP by electrostatic attraction and ion exchange, whereas CCBC-Mg and NZ-Mg, mainly relied on struvite precipitation on the material surface. The Mg2^+^ - modification increased the solution pH and formed Mg-P precipitates on the material surface, providing a favourable alkaline environment and nucleation conditions for the struvite reaction, thereby promoting the simultaneous recovery of AN and TP by the materials. This study could provide a theoretical basis for the design of struvite carrier for the simultaneous recovery of AN and TP from LPFE by the struvite precipitation method, and promote the application of this process in livestock and poultry farming effluent treatments.

## Introduction

With the consumption of dairy products and meat increasing significantly, livestock farming has become one of the fastest growing industries.Nevertheless, Pollution caused by the large amount of livestock and poultry farming effluents (LPFE) has become an indisputable concern [1]. According to the “Second National Pollution Source Census Bulletin of China,” in 2017, the livestock and poultry farming industry discharged more than 10 million tons of COD, more than 100,000 tons of AN, nearly 600,000 tons of total nitrogen, and more than 100,000 tons of TP into waterways, accounting for 93.8%, 51.3%, 42.14%, and 56.46% of total discharges, respectively [2]. The wastewater from livestock and poultry farming has become a significant source of pollution to water environment. The primary pollutants in livestock and poultry farming effluents are N and P, which are also essential nutrients necessary for crop growth [3,4]. Due to the characteristics of phosphorus, including its singular form, non-renewability, and limited reserves, humans primarily depend on the extraction of phosphate ores as the main source for obtaining phosphorus [5]. All over the world, about 1.3 Mt/a of phosphorus is immoderately discharged into ecosystem and the phosphate might be exhausted in 50 years [6], which will cause eutrophication and aggravate the risk of global food production [7]. Moreover, As a common nitrogen compound, ammonia nitrogen is directly discharged into the ecological environment, which will cause groundwater contamination, excessive algal growth, and water quality degradation. In response to the resource shortage and the environmental pollution, a highly effective and reliable nutrients recovery method is necessary to save resources and alleviate ecological stress.This subject has garnered significant attention within the domain of water treatment technology research. Struvite (MAP) is a crystalline mineral with the molecular formula MgNH4PO4·6H2O.Due to its high efficiency, ease of operation and environmental friendliness, the struvite process has been identified as one of the preferred chemical processes for the simultaneous recovery of nitrogen and phosphorus from wastewater.

The struvite product (MAP) recovered by this method is regarded as an ideal slow-release fertiliser [8] due to its high solubility (25°C, Ksp = 2.5 × 10 − 13). Research by Haiming [9] et al. demonstrated that this method can achieve approximately 93% recovery/removal efficiency of phosphates and AN from pig farm wastewater.Struvite precipitation has been widely adopted for the recovery of N and P from wastewater; however, its drawbacks, including high alkali consumption and small crystal size, cannot be overlooked [10]. The utilisation of alkaline porous carrier materials is imperative to enhance struvite crystallization and precipitation while maintaining an optimal pH value [11].

Previous research has shown that the utilisation of Mg2^+^ -modified nucleation materials as seed crystals to enhance the MAP (Magnesium Ammonium Phosphate) crystalization precipitation reaction constitutes a pivotal approach to curtailing alkali consumption and augmenting the particle size of MAP products. The selection of inexpensive, efficient, and suitable carriers for struvite crystallization is of significant practical significance. To resolve these bottlenecks, several studies [12,13] have used magnesium modified porous adsorbents as struvite carriers to integrate struvite deposits.

Natural zeolite is composed of silica tetrahedra and alumina tetrahedra and belongs to the silicate minerals. It has high selective adsorption for AN, but due to its negatively charged surface, it has almost no ability to remove P [12]. In order to enhance their capacity to remove AN, while concomitantly acquiring the ability to remove P [14], natural zeolites must undergo modification.After was modified by magnesium salt, the N and P removal capacity of natural zeolite was enhanced [15]. Corn cob, as a by-product of corn with an annual output of more than 40 million tons [15] and possesses the characteristics of high carbon content and natural fibre structure [16], which can be used as a raw material for biochar materials.Biochar has been identified as a potentially effective adsorbent for the removal of N and P nutrients [17]. However, most biochar have poor adsorption capacity for phosphorus due to their predominantly negative surface charge [18]. The adsorption capacity of phosphorus can be enhanced by modified biochar with magnesium [19]. Its application in treating pig wastewater has been demonstrated to result in the generation of struvite precipitate, a process that facilitates the recovery of N and P resources [20].

Despite the fact that both biochar and natural zeolite materials function as struvite carriers during simultaneous nitrogen and phosphorus recovery, there are discrepancies in their specific applications.As struvite is a by-product produced during the adsorption of N and P by biochar materials, it is retentioned within the biochar matrix [21–27]. The application of biochar, which is rich in N and P, to soil has been shown to reduce the amount of fertilizer required and to act as a slow-release fertilizer, thereby improving nutrient uptake and reducing N leaching [28–31]. This suggests that biochar, which is rich in N and P, has considerable potential for use in agriculture. N and P-rich zeolite has been shown to enhance soil physical and chemical properties, thereby improving nutrient utilisation efficiency [32]. This suggests that zeolite could be a valuable material in reducing soil C-N gas loss. However, it is important to note that N and P-rich zeolite does not function as a “slow-release fertilizer”, and the N present in zeolite is immediately available to soil microorganisms. This immediate availability can lead to an increase in nitrification in soil, so nitrification inhibitors are considered when N and P-rich zeolites are used as soil amendments or fertilizers [33,34]. The recovery of N and P resources through magnesium modification has been observed in both corncobs and natural zeolite, though the recovery mechanisms and differences in these processes remain to be thoroughly examined. This paper aims to address this knowledge gap by comparing the simultaneous recovery effect of two magnesium modified materials on N and P in LPFE.

The study systematically investigated the influence of magnesium modification on material properties and precipitate characteristics. These findings establish a theoretical foundation and experimental reference for engineering self-alkaline carrier materials designed to achieve simultaneous N and P recovery through struvite precipitation in LPFE. This research further advances the practical applicability of struvite crystallization processes for LPFE treatment, demonstrating their enhanced efficacy and environmental benefits.

## Materials and methods

The full name of the authority:Institute of Plant Nutrition, Resources and Environment, Beijing Academy of Agriculture and Forestry Sciences. This research does not involve any toxic substances or dangerous microorganisms. All the research experiments were carried out in public laboratories, so no permission is required.

### Materials

In the experiment, corn cobs were collected from Pinggu District, Beijing, and natural zeolite was purchased from a company in Zhengzhou, Henan. The raw materials were subjected to a washing process with deionized water, followed by drying to constant weight at 80 °C. Subsequently, corn cobs and natural zeolite were processed using a crusher. The corn cobs were selected to be 1 mm in size, while the natural zeolite was chosen to be 0.15 mm. The subject of the experiment was simulated LPFE, which was prepared using ammonium chloride, dipotassium hydrogen phosphate trihydrate, and deionized water with an AN content of 120 mg/L and a TP content of 12 mg/L. The initial AN, TP concentration, and pH of the simulated and actual LPFE are shown in Table 1. With the exception of magnesium chloride hexahydrate (MgCl_2_·6H_2_O), which was procured from Shanghai Aladdin Biochemical Technology Co., Ltd., all other reagents, including dipotassium hydrogen phosphate trihydrate (K_2_HPO_4_·3H_2_O), ammonium sulfate ((NH4)2SO4), potassium sodium tartrate (NaKC_4_H_4_O_6_), and concentrated sulfuric acid (H2SO4), were obtained from Sinopharm Group Chemical Reagent Co., Ltd. (analytical grade). All solutions were prepared using deionized water.

**Table 1:** Indicators of LPFE.

Wastewater types	NH4-N(mg/L)	TP(mg/L)	pH	Reference
Simulated wastewater	120	12	7.39	This paper.
Anaerobic digestion effluent from cow manure	100-700	10-60	7-10	Gong et al. 2018 [35]
Pig farm wastewater	n.a.	10-80	9	Wrigley et al. 1992 [36]
	106.73	10.62	6.8	Wu et al. 2024 [37]

### Preparation of CCBC-Mg and NZ-Mg

Mg2^+^ modified corn cob biochar and untreated corn cob biochar samples were prepared according to the following procedures. Firstly, 10 g corn cob was added into a 250 mL conical flask with 100 mL MgCl_2_·6H_2_O solution (1 mol/L). Then, The mixture was oscillated at a frequency of 120 rpm for a period of 12 hours in a thermostatic oscillator. The precipitate obtained was collected by filtration and washed with ultrapure water. Thirdly,the solid samples was placed in a drying oven set at 80°C until it reached a constant weight.the furnace was purged with 300 mL/min N_2_ for 30 minutes to remove all air.The solid sample was calcined in tubular furnace at 400 °C for 60 min with 10 °C/min heating rate.N_2_ atmosphere (300 mL/min) must be maintained throughout the pyrolysis process. Finally, The desired absorbents (CCBC-Mg) were obtained via washing and drying and sieving through 1 mm nylon screens. the resulting sample was labeled CCBC-Mg. Concurrently, an untreated biochar control (CCBC) was prepared using the same process as CCBC-Mg with the exception that deionized water was used in place of MgCl_2_ solution. The prepared materials were stored in a desiccator for use in subsequent experiments.

Mg^2+^ modified natural zeolite and untreated natural zeolite samples were prepared according to the following procedures. Firstly, 10 g natural zeolite was added into a 250 mL conical flask with 100 mL MgCl_2_·6H_2_O solution (1 mol/L).Then, NaOH solution with the ratio of OH^-^/Mg^2+^ of 2 was added into the mixture and oscillated at 120 rpm for 12 hours in a thermostatic vibrator. Thirdly, The mixture was weighed, after which it was placed in a drying oven set at 80°C until it reached constant weight. The solid sample was calcined in muffle at 400 °C for 4hours with 10 °C/min heating rate. Finally, The desired absorbents (NZ-Mg) were obtained via washing and drying and sieving through 1 mm nylon screens.Concurrently, an untreated natural zeolite (NZ) was prepared using the same process as NZ-Mg with the exception that deionized water was used in place of MgCl_2_ solution. The prepared materials were stored in a desiccator for use in subsequent experiments.

### Adsorption experiments

Adsorption capacity comparison experiment The simultaneous recovery effect of N and P from simulated wastewater by various materials (CCBC, CCBC-Mg, NZ, NZ-Mg) was evaluated through three independent sets of parallel experiments. According to the different nucleation materials, each batch of experiments was grouped, and the recovery rate and adsorption capacity of each treatment were comprehensively analyzed. Firstly, 80 mg of the experimental material was precisely transferred into a 250 mL conical flask, then 80mL of simulated wastewater was added and stirred for 12 hours at ambient temperature(25°C) thermostatic vibrator at 120 rpm. Wastewater samples were systematically collected at predetermined intervals post-reaction initiation (60, 120, 240, 480, and 720 minutes). After the Wastewater samples were filtered by 0.45μm filter membrane, various indexes of the filtrate were determined, including pH, AN and TP concentration. The recovery rate (R, %) and adsorption capacity (Qe, mg/g) of N and P in Simulated wastewater by nucleating materials can be calculated by the following formula:


$$R(\%)=(C_{0}-C_{e})/C_{0}\times 100\%$$ (1)

$$Q_{e}=(C_{0}-C_{e})\cdot V/m$$ (2)

Where R (%) is the recovery rate of N and P from the simulated wastewater by the nucleation material, where C_e_(mg/L) and C_0_(mg/L) is the equilibrium concentration and initial concentration, respectively; V(mL) is the volume of the simulated wastewater; and m(g) is the mass of nucleation material added. The N and P indicators are based on the measurement of AN and TP in the aqueous solution.

Adsorption kinetics The experiment utilized simulated wastewater with consistent reagent composition containing 120 mg/L ammonia nitrogen (AN) and 24 mg/L total phosphorus (TP). Triplicate samples (40 mg each) of nucleation adsorption materials(NZ,NZ-Mg,CCBC and CCBC-Mg)were precisely measured and introduced into 100 mL centrifuge tubes containing 40 mL of the simulated wastewater. Initial solution parameters were controlled at pH 9 and N:P molar ratio of 5 for all experimental replicates. Secondly, The centrifuge tubes were oscillated in a oscillator under continuous agitation at ambient temperature (25°C) and 150 rpm for 24 hours. Wastewater samples were systematically collected at predetermined intervals post-reaction initiation (0, 30, 60, 120, 180, 360, 480, 720, and 1440 minutes). Finally, the samples were centrifuged at 12000 rpm for 5 min and filtered by 0.45 μm filter membrane to determine the concentrations of AN and TP in the filtrate.Scatter plots were drawn with adsorption capacity as the y-axis and sampling time as the x-axis, and the data were fitted using the pseudo-first-order and pseudo-second-order kinetic models to analyze and elucidate the adsorption principles of N and P by the nucleation materials NZ, NZ-Mg, CCBC, and CCBC-Mg. The pseudo-first-order and pseudo-second-order kinetic models are shown in Equations (3) and (4), respectively:


$$q_{e}=q_{t}(1-e^{k_{1}t})$$ (3)

$$\frac{t}{q_{t}}=\frac{1}{k2qe^{2}}+\frac{t}{q_{e}}$$ (4)

Where qt(mg/g) and qe(mg/g)are the adsorption capacity at time t and the adsorption capacity at equilibrium, respectively; k₁(min ⁻ ¹) and k₂(g/(mg·min)) are pseudo-first-order and pseudo-second-order reaction rate constants, respectively.

Adsorption isotherms Three portions (for parallel experiments) of 40 mg of nucleation adsorption materials (NZ, NZ-Mg, CCBC, and CCBC-Mg) were accurately weighed and added to 50 mL centrifuge tubes, Subsequently, 40 mL of standard solutions were added to each tube, containing gradient concentrations of ammonia nitrogen (AN: 10, 25, 50, 100, 200, 250, 300, 400 mg/L) and total phosphorus (TP: 4.4, 11, 22, 44, 88, 110, 132, 176 mg/L), respectively. The centrifuge tubes were oscillated in a thermostatic oscillator under continuous agitation at ambient temperature (25°C) and 150 rpm for 24 hours. The suspensions were allowed to settle undisturbed for 30 minutes. Finally, The samples were centrifuged at 12000 rpm for 5 minutes and filtered through a 0.45 μm filter membrane to measure the concentrations of AN and TP in the filtrate. Adsorption isotherms were constructed using equilibrium adsorption capacities corresponding to measured AN and TP concentrations.Isothermal adsorption models (Langmuir and Freundlich) were applied to the experimental data, with derived parameters subsequently employed to investigate the adsorption mechanisms governing nitrogen and phosphorus removal by these materials. The formulations of the Langmuir and Freundlich models are provided in Equations (5) and (6), respectively:


$$Q_{e}=\frac{k_{L}Q\max C_{e}}{1+C_{e}}$$ (5)

$$Q_{e}=k_{F}{C_{e}}^{1/n}$$ (6)

Where Qe (mg/g) and Qmax (mg/g) are the equilibrium and maximum adsorption capacities, respectively; Ce (mg/L) is the concentration at equilibrium; kL (L/mg) and kF (mg/g(L/mg)1/n) are the Langmuir and Freundlich constants, respectively.

### Characterizations

1.0 g of unmodified materials (CCBC and NZ) and Mg^2+^ -modified materials (CCBC-Mg and NZ-Mg) were individually introduced into 1000 mL aliquots of livestock wastewater (2000 mg/L AN、200 mg/L TP). The mixtures were incubated at 25°C under constant agitation (120 r/min) for 8 hours using an thermostatic vibrator. Then the mixed sample was taken, centrifuged at 20000 r/min for 5 minutes, and the supernatant was recovered. The crystalline precipitate products of the remaining mixed sample were subjected to vacuum filtration. The filtered products were dried in a 40 °C dryer to a constant weight, and then the characterization and analysis of the precipitate products were carried out. The precipitate products formed after the unmodified material adsorbed nitrogen and phosphorus were recorded as CCBC-P and NZ-P. The precipitate products formed after magnesium modified materials adsorb nitrogen and phosphorus are denoted as CCBC-Mg-P and NZ-Mg-P.

The specific surface area and pore size distribution of the material before and after modification were determined using a Brunauer-Emmett-Teller analyzer (BET) (Hitachi S-4800, Japan). The microstructure of the materials and the corresponding precipitates before and after modification was observed using a scanning electron microscope (SEM) (Fei Inspect F50, USA). The changes in functional groups on the surface of the materials and the corresponding precipitates before and after modification were detected using a Fourier transform infrared spectroscopy analyzer (FTIR) (Nicolet iS5, USA). The crystal structures of the material surface and the corresponding precipitates were determined using X-ray powder diffraction (XRD) (Bruker D8 ADVANCE, Germany)analysis before and after modification.

### Analytical methods

The concentrations of AN and TP were determined using the Nessler’s reagent spectrophotometric method and the ammonium molybdate spectrophotometric method, respectively, with a spectrophotometer (DR6000, USA). The pH value was measured using a pH meter (FiveEasy Plus, Switzerland).

### Data analysis

The data processing and graphing were conducted utilising Microsoft Excel, Origin 2021, and SPSS software. The statistical analysis of the data was performed using one-way analysis of variance (ANOVA), and post hoc mean comparisons were conducted using the Tukey test (p < 0.05, p < 0.01). Error bars represent the standard error (SE), calculated from the experiments performed in triplicate.

ANOVA analysis of variance is used to analyze whether there are significant differences in the means of multiple groups of samples under the influence of a single control variable. The Turkey test (Tukey test) is a statistical method used for multiple comparisons following Analysis of Variance (ANOVA), mainly to determine significant differences among the means of different groups. The core lies in achieving reliable analysis through multiple pairwise comparisons by controlling the overall error rate.

## Results and discussion

### Adsorption experiments

**Fig 1:**
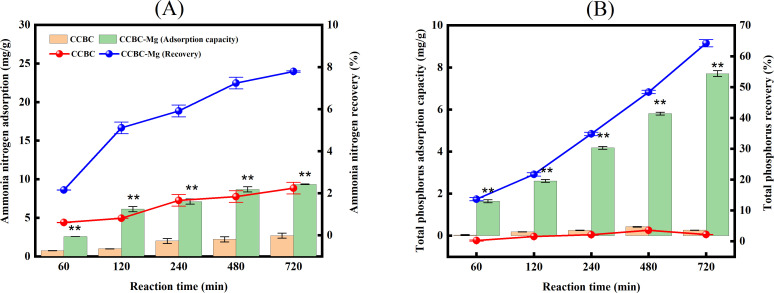
AN and TP adsorption capacity and recovery rate of corncob biochar-based materials. (A): AN. (B): TP.

Effect of Mg2^+^ -modification on the recovery of AN and TP from LPFE by corn cob biochar materials A comparison of the N adsorption capacities and recovery rates between CCBC-Mg and CCBC is demonstrated in Fig 1a. The results demonstrated that with an increase in reaction time, both the adsorption capacity and recovery rate of AN for CCBC-Mg and CCBC exhibited an upward trend. The adsorption capacities for AN were found to be 2.59 ~ 9.34 mg/g and 0.73 ~ 2.68 mg/g for the two treatments, respectively, with recovery rates reaching 2.16% ~ 7.74% and 0.61% ~ 2.24%, respectively. At each reaction times of 60, 120, 240, 480, and 720 minutes, the AN adsorption capacity of CCBC-Mg was significantly higher than that of CCBC (p < 0.01), with the magnesium-modified corn cob biochar exhibiting an increase of 1.54% ~ 5.57% in AN adsorption capacity compared to the unmodified corn cob biochar. A comparison of the TP adsorption capacities and recovery rates between CCBC-Mg and CCBC is shown in Fig 1b. The results indicated that as the reaction time increased, both the adsorption capacity and recovery rate of TP for CCBC-Mg showed an upward trend, while those for CCBC initially increased and then decreased. The adsorption capacities for TP were found to range from 1.64 to 7.70 mg/g and from 0.04 to 0.26 mg/g for the two treatments, respectively, with recovery rates reaching 13.28% to 63.26% and 0.30% to 2.17%, respectively. The findings indicate that the absence of Mg^2+^ modification in the corn cob biochar (CCBC) renders it devoid of P adsorption capability, underscoring the pivotal role of magnesium salt pretreatment in enhancing the P adsorption performance of corn cob biochar materials. At each reaction times of 60, 120, 240, 480, and 720 minutes, the TP adsorption capacity of CCBC-Mg was significantly higher than that of CCBC (p < 0.01), with the magnesium-modified corn cob biochar exhibiting an increase of 13.58% ~ 61.09% in TP adsorption capacity compared to the unmodified corn cob biochar. These results are consistent with the conclusions of scholar Wan S [38], who found that biochar modified with magnesium significantly increased its adsorption capacity and recovery rate for P.

**Fig 2:**
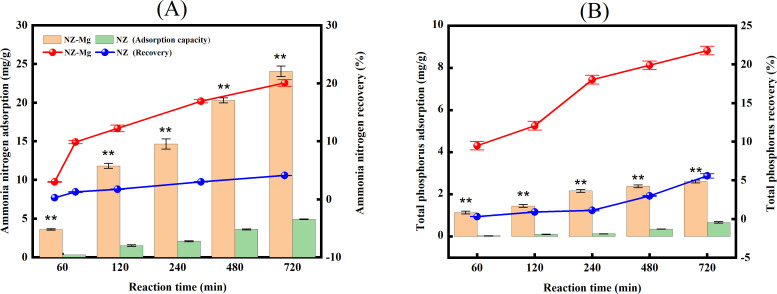
The AN and TP adsorption capacities and recovery rates of natural zeolite-based materials. (A): AN. (B): TP.

Effect of Mg^2+^ -modification on recovery of AN and TP from LPFE by natural zeolite materials A comparison of the AN adsorption capacity and recovery rate between NZ-Mg and NZ is shown in Fig 2a. The results demonstrated that with an increase in reaction time, both NZ-Mg and NZ exhibited an enhancement in AN adsorption capacity and recovery rate. The AN adsorption capacities for the two treatments were 3.59 ~ 24.04 mg/g and 0.33 ~ 4.92 mg/g, respectively, with corresponding recovery rates of 2.99% ~ 20.04% and 0.28% ~ 4.10%. At each reaction times of 60, 120, 240, 480, and 720 minutes, the AN adsorption capacity of NZ-Mg was significantly higher than that of NZ (p < 0.01), with the magnesium-modified zeolite exhibiting an increase of 2.61% ~ 15.94% over the unmodified zeolite. The comparison of the TP adsorption capacity and recovery rate between NZ-Mg and NZ is demonstrated in Fig 2b. The results indicated that as the reaction time increased, both NZ-Mg and NZ exhibited an upward trend in TP adsorption capacity and recovery rate. The TP adsorption capacities for the two treatments were 1.13 ~ 2.61 mg/g and 0.03 ~ 0.67 mg/g, respectively, with corresponding recovery rates of 9.48% ~ 21.78% and 0.29% ~ 5.57%. At the commencement of the reaction, the zeolite materials demonstrated diminished P adsorption capacities, attributable to the pH of the NZ treatment reaction system, which ranged from 7.76 to 8.14. When the solution pH was between 7.20 and 12.33, P exists in the form of HPO_4_^2-^ [39].The hydroxyl and other functional groups on the surface of the NZ samples exhibited an enhanced deprotonation trend, resulting in a more negative surface Zeta potential. This, in turn, generates electrostatic repulsion with the negatively charged HPO_4_^2-^ in the water, leading to lower P adsorption capacities [40]. At each reaction times of 60, 120, 240, 480, and 720 minutes, the TP adsorption capacity of NZ-Mg was significantly higher than that of NZ (p < 0.01), with the magnesium-modified zeolite exhibiting an increase of 9.19% ~ 16.21% over the unmodified zeolite.The novel magnesium oxide/iron oxide-modified NaY zeolite (MNZ) developed by scholar Zhenwei Lu [13] exhibits high adsorption performance for both AN and phosphate, which is consistent with the conclusions of this study.

**Fig 3:**
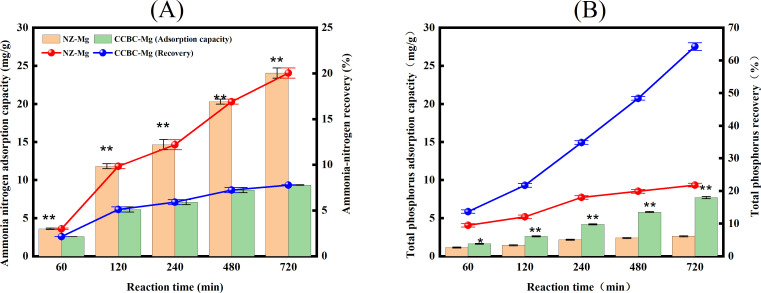
The AN and TP adsorption capacities and recovery rates of magnesium-modified materials. (A): AN. (B): TP.

Comparison of effect of different Mg2^+^ -modified materials on the recovery of AN and TP from LPFE A comparison of the AN adsorption capacity and recovery rate between NZ-Mg and CCBC-Mg is shown in Fig 3a. At the 60-minute reaction time, the AN adsorption effect of NZ-Mg was significantly higher than that of CCBC-Mg (p < 0.05). Furthermore, at each reaction times of 120, 240, 480, and 720 minutes, the AN adsorption capacity of the NZ-Mg treatment was found to be extremely significantly higher than that of the CCBC-Mg treatment by 0.85% to 12.25% (p < 0.01). The comparison of the TP adsorption capacity and recovery rate between NZ-Mg and CCBC-Mg is demonstrated in Fig 3b. The findings suggest that at the 60-minute reaction time, the TP adsorption effect of CCBC-Mg was significantly higher than that of NZ-Mg (p < 0.05). Furthermore, at each reaction times of 120, 240, 480, and 720 minutes, the TP adsorption capacity of the CCBC-Mg treatment was found to be extremely significantly higher than that of the NZ-Mg treatment by 4.19% to 42.43% (p < 0.01). These findings indicate that CCBC-Mg is more effective in the recovery of P from the wastewater, while NZ-Mg is more effective in the recovery of AN from the wastewater.

**Fig 4:**
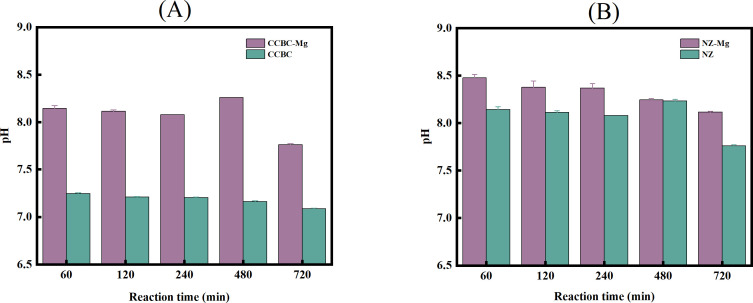
Trend chart of pH changes during the adsorption process of N and P from LPFE by materials. (A) Corncob biochar-based material. (B) Natural zeolite-based material.


Effect of magnesium modification on pH As demonstrated in Fig 4, the pH values of the two modified materials (CCBC-Mg and NZ-Mg)were notably higher, ranging from 8.16–8.67 and 8.11–8.51, respectively. These values represent a significant increase compared to their unmodified counterparts(CCBC and NZ), which exhibited pH ranges of 7.08–7.24 and 7.76–8.14, respectively. This substantial increase in pH can be attributed to the reaction of Mg present on the material surface with water, producing Mg(OH)₂ and releasing OH ⁻ , which effectively raised the pH of the solution [41,42]. The resulting alkaline environment is particularly beneficial, as it enhances the removal of AN and TP by CCBC-Mg and NZ-Mg [43]. Furthermore, formula (7) demonstrates that the presence of N、P, and Mg² ⁺ triggers the struvite precipitation reaction within the system. This reaction further contributes to the removal of these nutrients from the solution, underscoring the effectiveness of the Mg² ⁺ -modified materials in nutrient management.

$$Mg^{2+}+NH4^{+}+6H_{2}O+HnP{O_{4}}^{3-}\to MgNH_{4}PO_{4}\cdot 6H_{2}O+nH^{+}$$ (7)

This process facilitates the removal of N and P from the system [17], as alkaline conditions are known to be more favorable for driving the struvite precipitation reaction towards completion, thereby shifting the chemical equilibrium to the right. Notably, the pH of the solutions containing Mg² ⁺ -modified materials (CCBC-Mg, NZ-Mg) demonstrated a negative correlation with time. This gradual decrease in pH is attributed to the release of H⁺ during the struvite precipitation process, which occurs concomitantly with the recovery of N and P, as evidenced by the experimental results.

**Fig 5:**
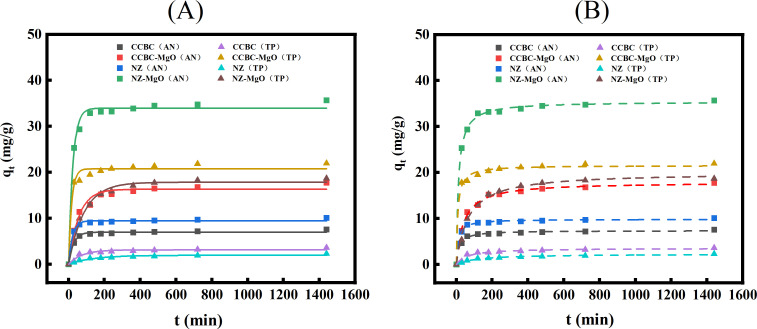
Kinetic models for material adsorption of AN and TP from LPFE. (A): Pseudo-first-order kinetic model. (B): Pseudo-second-order kinetic model.

Adsorption kinetics As demonstrated in Fig 5, the adsorption rates of AN and TP by the four materials were initially rapid during the first 120 minutes, followed by a gradual deceleration until equilibrium was reached at 360 minutes.At equilibrium, the adsorption capacities for AN and TP across all four materials remained relatively stable over time. As presented in Table 2, the kinetic models for CCBC, CCBC-Mg, NZ, and NZ-Mg all exhibited high R² values, suggesting that both pseudo-first-order and pseudo-second-order models effectively describe the adsorption of nitrogen and phosphorus by these materials. Notably,the pseudo-second-order model demonstrated superior R² values compared to the pseudo-first-order model, with the exception of phosphorus recovery by CCBC material. This observation implies that while physical adsorption processes contribute to AN and TP removal, the dominant mechanism is chemical adsorption, including ion exchange, chemical precipitation, and ligand exchange [15,44–46]. In conjunction with BET analysis results, these findings reinforce that the enhanced adsorption capacity of modified materials (CCBC-Mg, NZ-Mg) for AN and TP is not solely attributed to their specific surface area, pore size, or pore volume. Instead, the adsorption process for unmodified materials (CCBC, NZ) is primarily governed by the formation of chemical bonds through electron transfer, exchange, or sharing between NH₄ ⁺ , PO₄³⁻ and HPO₄²⁻ in solution and surface functional groups [47,48]. The limited TP adsorption capacity of zeolite can be attributed to its electrostatic interactions and ion exchange properties, which preferentially adsorb cations like NH₄ ⁺ rather than PO₄³⁻ [49]. Moreover, magnesium compounds in modified materials (CCBC-Mg, NZ-Mg) release Mg²⁺ into solution, facilitating Mg-P precipitate formation [50]. Furthermore, the low surface energy of newly formed phosphate precipitates promotes nucleation and growth on the material surface [51], enhancing overall adsorption efficiency.

**Table 2:** Fitting parameters for adsorption kinetics of AN and TP.

Materials	Type of nutrient recovered	Pseudo-first-order			Pseudo-second-order		
		k_1_ (min^-1^)	q_e_ (mg/g)	R^2^	k_2_ (min^-1^)	q_e_ (mg/g)	R^2^
CCBC	AN	0.035	6.98	0.984	0.008	7.37	0.992
CCBC-Mg		0.017	16.31	0.972	0.001	17.92	0.995
NZ		0.045	10.06	0.991	0.009	9.83	0.996
NZ-Mg		0.040	34.25	0.973	0.002	35.90	0.993
CCBC	TP	0.013	3.13	0.954	0.004	3.55	0.945
CCBC-Mg		0.056	20.773	0.975	0.005	21.54	0.992
NZ		0.017	1.950	0.954	0.003	2.28	0.974
NZ-Mg		0.012	17.83	0.991	0.007	20.02	0.994

**Fig 6:**
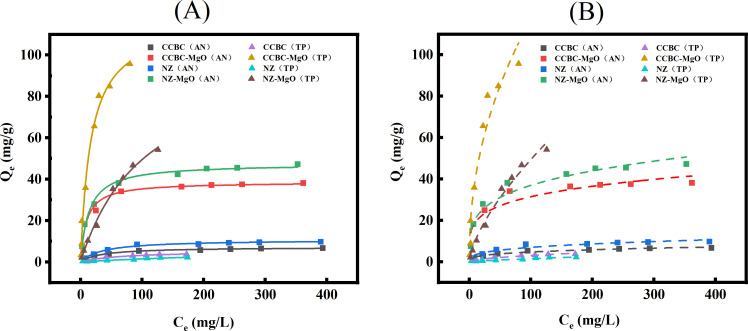
Isothermal adsorption models for AN and TP adsorption from LPFE using various materials. (A): Langmuir model. (B): Freundlich model.

Adsorption isotherm As illustrated in Fig 6 and Table 3, the adsorption isotherm curves for AN and TP demonstrated excellent agreement with both the Langmuir and Freundlich models across all four materials, yielding R² values exceeding 0.95. This indicates that the adsorption process is governed by a combination of physical and chemical adsorption, with chemical adsorption being the dominant mechanism. The marginally higher R² values obtained for the Langmuir model imply that the adsorption of AN and TP by the materials primarily follows a monolayer homogeneous adsorption process [52]. Based on the Langmuir model, the maximum adsorption capacities for AN followed the order: NZ-Mg (47.50 mg/g)> CCBC-Mg (38.66 mg/g)> NZ (10.72 mg/g)> CCBC (7.15 mg/g). For TP, the order was: CCBC-Mg (116.99 mg/g)> NZ-Mg (103.11 mg/g)> CCBC (8.45 mg/g)> NZ (5.38 mg/g). Overall, zeolite materials (NZ, NZ-Mg) exhibited superior AN adsorption capacities, whereas corncob biochar materials (CCBC-Mg, CCBC) demonstrated enhanced TP adsorption capabilities. The Freundlich isotherm parameter kF, which reflects the material’s adsorption capacity for N and P, revealed that higher k_F_ values correlate with stronger nutrient recovery intensities. These findings align with previous studies [38,44], confirming that Mg² ⁺ -modification significantly enhances the maximum adsorption capacities of both natural zeolites and biochar-based materials for N and P. This improvement stems from the presence of NH₄⁺ and PO₄³⁻ in wastewater, enabling individual adsorption of N and P by the materials. Additionally, Mg² ⁺ -modified surfaces develop a honeycomb-like porous structure due to Mg particle aggregation, providing abundant attachment sites for N and P enrichment. Surface-loaded Mg undergoes in-situ hydrolysis, generating Mg(OH)₂ intermediates at the solid-liquid interface, which subsequently dissolve to release Mg²⁺ and OH⁻ into the solution [53]. This process creates a favorable environment for struvite formation, significantly augmenting the adsorption capacities for AN and TP. The Freundlich model further characterized adsorption intensity using the parameter n, where n < 1, 1 < n < 2, and 2 < n < 10 indicate poor, moderate, and good adsorption, respectively [54]. Experimental results support this model, showing that CCBC-Mg, NZ, and NZ-Mg exhibit good AN adsorption, while CCBC demonstrates moderate adsorption. Similarly, CCBC-Mg and NZ-Mg exhibit good TP adsorption, whereas CCBC and NZ show moderate TP adsorption. These findings are consistent with previous reports by Ronghua Li [19] and Haiming Huang [49].

**Table 3:** Fitting parameters for isothermal adsorption of AN and TP.

Materials	Type of nutrient recovered	Langmuir			Freunlich		
		k_L_ (L/mg)	Q_m_ (mg/g)	R^2^	kF (mg/g (L/mg)^1/n^)	n	R^2^
CCBC	AN	0.027	7.15	0.981	1.18	1.33	0.961
CCBC-Mg		0.105	38.66	0.980	7.63	4.63	0.952
NZ		0.028	10.72	0.986	1.643	3.20	0.953
NZ-Mg		0.076	47.50	0.989	11.67	3.99	0.974
CCBC	TP	0.014	5.38	0.972	0.235	1.80	0.954
CCBC-Mg		0.059	116.99	0.987	13.95	2.16	0.975
NZ		0.002	8.45	0.971	0.032	1.21	0.963
NZ-Mg		0.009	103.11	0.994	9.75	2.48	0.982

### Characterization analysis

BET analyses As presented in Table 4, the specific surface area, pore diameter, and pore volume of CCBC-Mg exhibit 1.19, 1.61, and 1.40 fold increases, respectively, compared to its unmodified CCBC counterpart. This substantial enhancement confirms the effective role of Mg^2+^ -modification in promoting pore structure development within CCBC-Mg.In contrast, NZ-Mg demonstrates 0.744, 0.844 and 0.835 fold reductions in specific surface area, pore diameter, and pore volume, respectively, relative to unmodified NZ. These decreases indicate that Mg²⁺ modification did not improve, and in fact slightly diminished, the pore characteristics of NZ-Mg compared to its zeolite precursor.Notably, the zeolite materials (NZ, NZ-Mg) exhibit markedly higher specific surface areas and pore volumes than their corncob biochar counterparts (CCBC, CCBC-Mg). This observation underscores the inherently superior porous architecture and surface area characteristics of zeolite materials, which contribute to their established efficacy in adsorption applications. The data collectively highlight material-specific responses to Mg^2+^ -modification and inherent structural differences between zeolite and biochar matrices.

**Table 4:** Pore structure parameters.

Materials	Specific surface area (m^2^/g)	Aperture (nm)	Pore volume (cm^3^/g)
CCBC	2.02	7.33	0.0042
CCBC-Mg	2.41	11.83	0.0059
NZ	14.60	11.24	0.067
NZ-Mg	19.62	13.31	0.056

**Fig 7:**
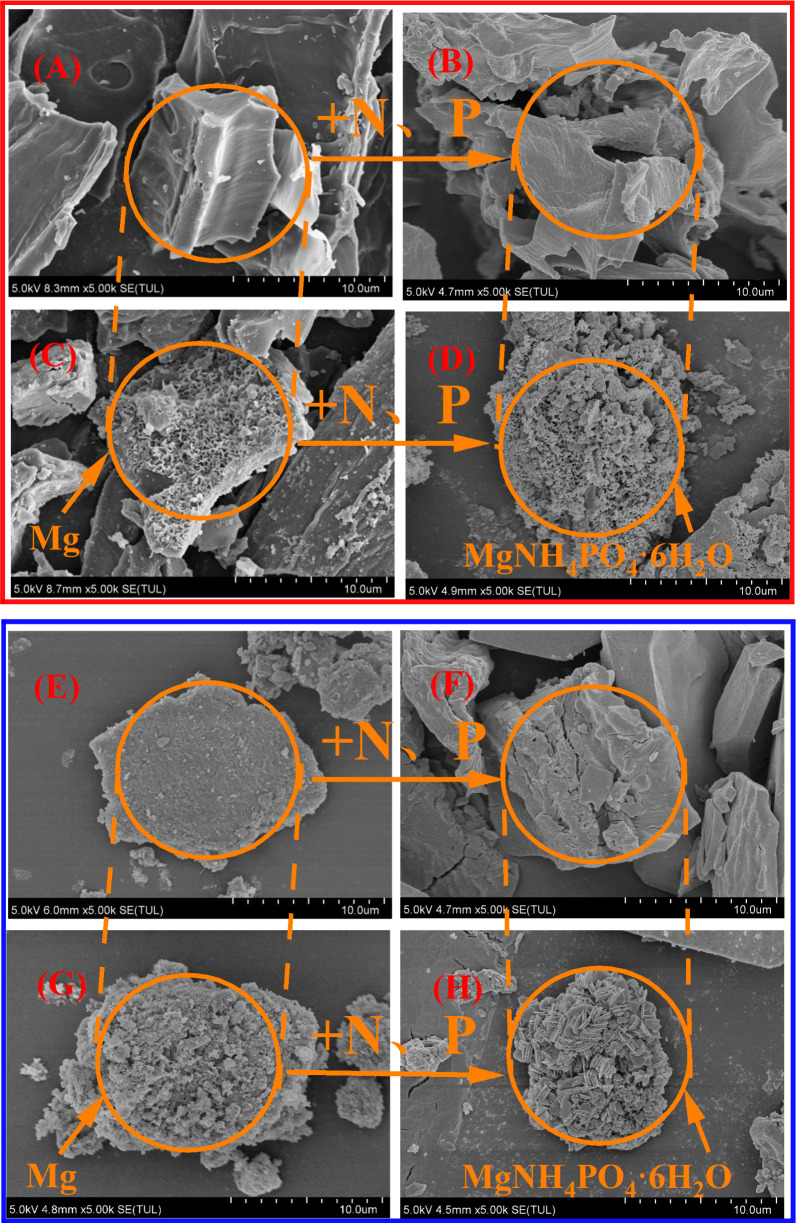
SEM images of of CCBC (A), NZ(B), CCBC-Mg(C), NZ-Mg(D) and the corresponding precipitated products CCBC-P(E), NZ-P(F), CCBC-Mg-P(G), NZ-Mg-P(H) (10.0 μm).

**Fig 8:**
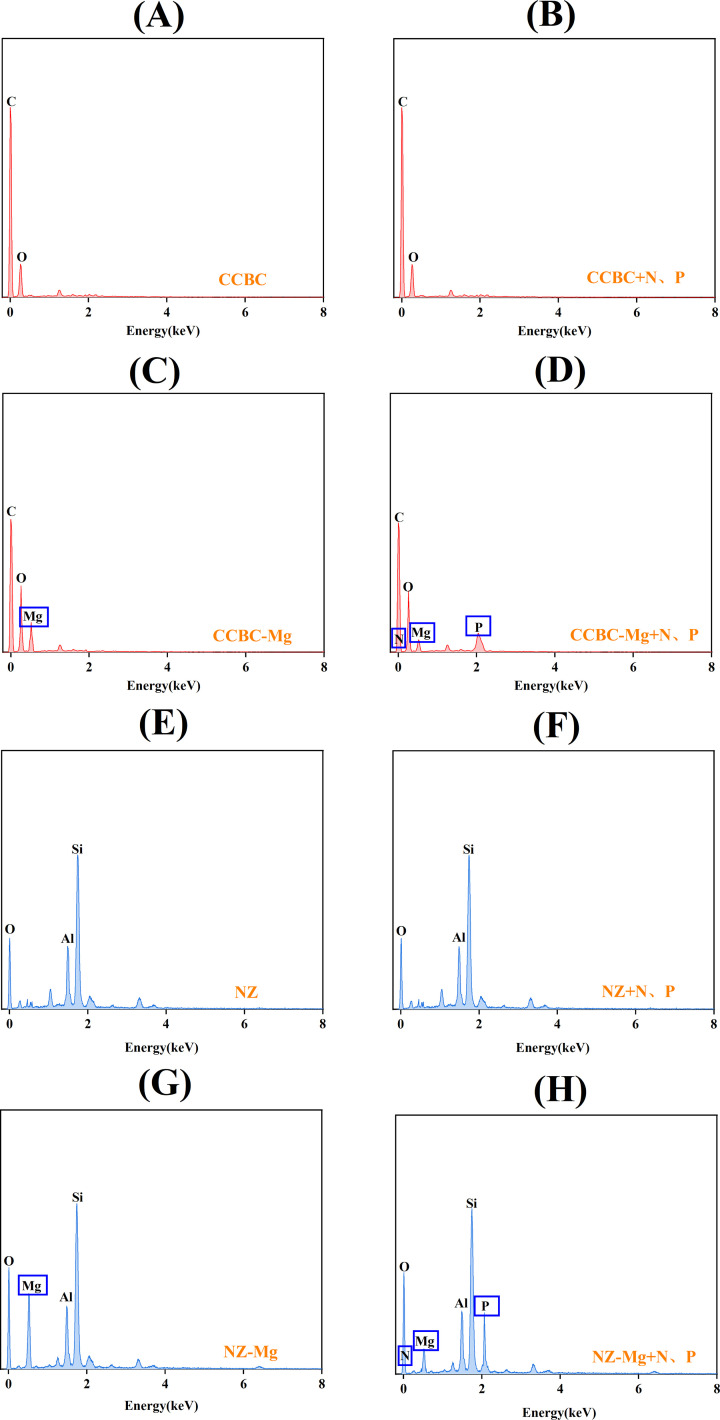
EDS images of CCBC (A), NZ(B), CCBC-Mg(C), NZ-Mg(D) and the corresponding precipitated products CCBC-P(E), NZ-P(F), CCBC-Mg-P(G), NZ-Mg-P(H).

SEM and EDS analyses The microstructure and element content of corncob biochar before and after modification are shown in Fig 7(A),(C), Fig 8(A),(C) and Table 5. The surface structure of CCBC is relatively smooth. The surface structure of CCBC is relatively smooth. The corresponding EDS analysis showed that CCBC surfaces contained large amounts of C(74.63%) and O(16.70%). However, the surface of CCBC-Mg is rough and tightly covered by a large number of sheet-like crystals, which are stacked to form a honeycomb-like structure. CCBC – Mg surface C content (74.63% ~ 49.89%), O (16.70% ~ 29.71%) and Mg (0.04% ~ 21.42%) content increased significantly; The microstructure and element content of natural zeolite before and after modification are shown in Fig 7(B),(D), Fig 8(B),(D) and Table 5. The surface structure of NZ is relatively flat, and there are few particles or flaky materials attached. The corresponding EDS analysis showed that the NZ surface contained a large amount of Si(53.42%) and a small amount of O(9.17%) and Al(6.23%). The surface of NZ-Mg has a flocculent coating, and the distribution is relatively uniform. NZ – Mg surface C content (53.42% ~ 49.61%), no obvious changes, O (9.17% ~ 14.72%) and Mg (0.03% ~ 15.19%) content increased significantly. The results are consistent with FTIR spectra, which proves the successful loading of Mg.

**Table 5:** Elemental composition of the material.

Materials	Element content by EDS (Weight)						
	Mg(%)	C(%)	O(%)	Al(%)	Si(%)	P(%)	N(%)
CCBC	0.04	74.63	16.70	n.a	n.a	0.00	0.01
CCBC-P	0.04	73.82	15.89	n.a	n.a	0.13	0.21
CCBC-Mg	21.42	49.89	29.71	n.a	n.a	0.01	0.01
CCBC-Mg-P	3.19	23.89	22.87	n.a	n.a	10.81	7.56
NZ	0.04	n.a.	9.17	6.32	53.42	0.00	0.03
NZ-P	0.03	n.a.	11.23	6.98	52.39	0.16	0.19
NZ-Mg	15.19	n.a.	14.72	5.74	49.61	0.00	0.02
NZ-Mg-P	2.91	n.a.	7.23	5.55	47.37	8.83	9.72

The microstructure and element content of corncob biochar before and after N and P adsorption are shown in Fig 7(A),(B),(C),(D), Fig 8(A),(B),(C),(D) and Table 5. The surface structure of CCBC-P did not change significantly, and the corresponding EDS analysis showed that the surface C content (74.63% ~ 73.82%), O content (16.70% ~ 15.89%) and Mg content (0.04% ~ 0.05%) did not change significantly. The surface of CCBC-P adsorbed very little N(0.13%) and P(0.21%). A large number of rod-like crystals are attached to the surface of CCBC-Mg-P. The corresponding EDS analysis showed that the Mg content (21.42% ~ 3.19%) on the CCBC-Mg-P surface decreased significantly, while the N content (0% ~ 7.65%) and P content (0.01 ~ 10.81%) increased significantly, indicating that the Mg on the surface was consumed to produce struvite precipitate during the nitrogen and phosphorus recovery reaction; The microstructure and element content of natural zeolite materials before and after N and P adsorption are shown in Fig 7(E),(F),(G),(H), Fig 8(E),(F),(G),(H) and Table 5. NZ-P surface structure has no obvious change, the EDS analysis showed that surface of Si content (53.42% ~ 52.39%), O content (9.17% ~ 10.01%), Al content (6.32% ~ 6.98%), and Mg content (0.03% ~ 0.03%) no significant change. A large number of rod-like or flaky crystals are attached to the surface of NZ-Mg-P. The corresponding EDS analysis showed that the Mg content (15.19% ~ 2.91%) on the NZ-Mg-P surface decreased significantly, while the N content (0% ~ 8.83%) and P content (0.02% ~ 9.72%) increased significantly, indicating that the Mg on the surface was consumed in the nitrogen and phosphorus recovery reaction to produce a guanite precipitated. The results were consistent with FTIR spectra, which proved the formation of struvite precipitation.

**Fig 9:**
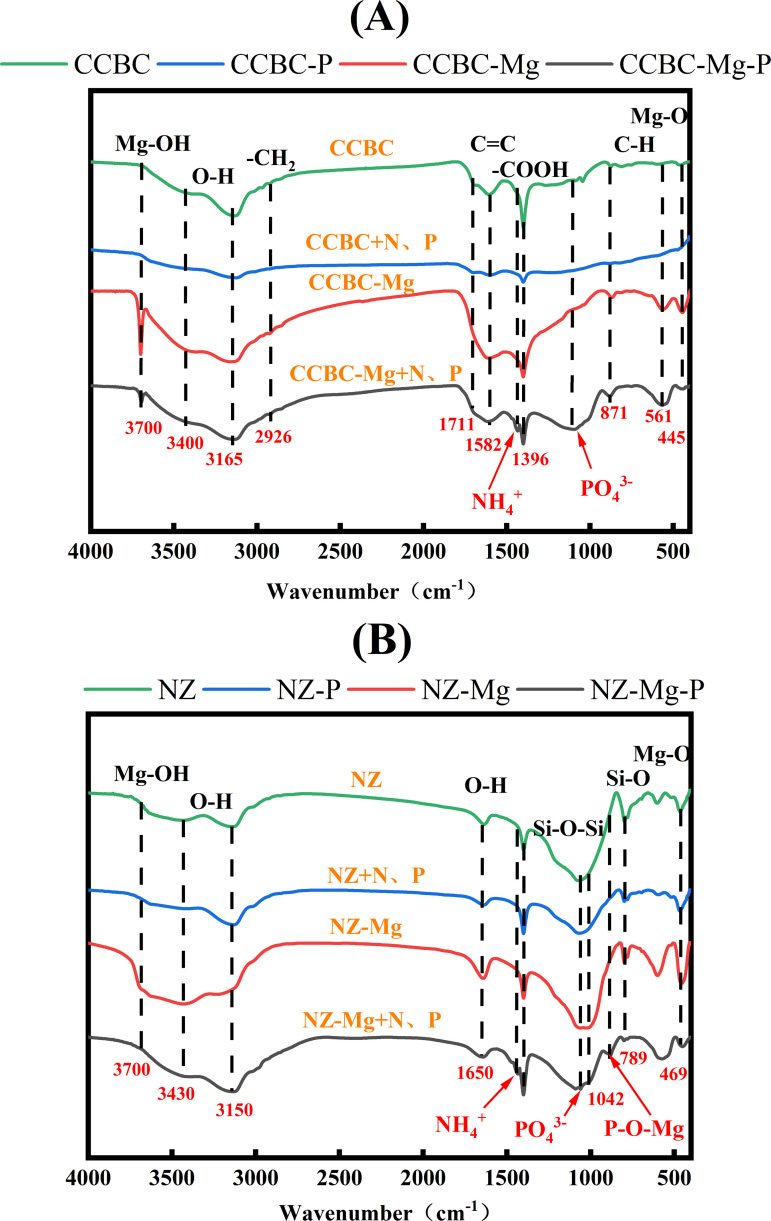
FTIR images. (A): Corncob biochar-based materials. (B): Natural zeolite-based materials.

FTIR analyses The FTIR spectra of corncob biochar before and after modification are presented in Fig 9(A), While the spectral characteristics of CCBC and CCBC-Mg were analogous, the intensity of specific absorption peaks varied significantly, indicating differences in the numbers of surface functional groups between the two samples. In comparison with CCBC, CCBC-Mg demonstrates Mg-O stretching vibration peaks and Mg-OH stretching vibration peaks at 445 cm-1 and 3700 cm-1, respectively [55]. These spectral characteristics are in agreement with the previously discussed SEM characterization results, confirming effective immobilization of Mg onto the biochar surface. The occurrence of stable aromatic hydrocarbon frameworks in both biochar types is evidenced by the presence of C = C stretching vibrational peaks near 1582 cm ⁻ ¹ [56]. The absorption bands observed within the wavenumber range of 500–900 cm ⁻ ¹ are attributed to the aromatic C–H stretching vibrational modes [57]. Notably, the intensified absorption peak at 561 cm^-1^ and 871 cm ⁻ ¹ observed in CCBC-Mg indicates that the modification process enhances the aromatic character of the carbonaceous material [58]. The bands at 1711/1396, 3165/3400 and 2926cm^ − 1^ prove the existence of carboxylic C (-COOH), phenolic O-H and aliphatic -CH2 stretch, respectively.comparative analysis of CCBC and CCBC-Mg spectra demonstrates reduced peak intensities at 3165/3400 cm-1, 2926 cm ⁻ ¹, and 1396 cm ⁻ ¹, alongside an augmented intensity at 561 cm ⁻ ¹ and 871 cm-1 [59–61]. These spectral alterations suggest a diminution of hydroxyl and carboxyl functional groups, counterbalanced by an elevation in carbonyl functionalities post-modification. Such transformations enhance the biochar’s proficiency in N and P adsorption from aqueous solutions. This observation aligns with prior research conclusions that attribute various adsorption phenomena to carbonyl surface functionalities formed during low-temperature biomass pyrolysis [62].

The FTIR spectra of Natural zeolite before and after modification are presented in Fig 9(B). The spectral profiles of Mg² ⁺ -modified NZ-Mg and pristine zeolite NZ exhibited comparable features, indicating that the zeolite’s inherent framework remained intact after modification. This suggests that Mg² ⁺ incorporation had negligible impact on the zeolite’s structural integrity. The spectral region between 3150 and 3430 cm ⁻ ¹ corresponds to O-H stretching vibrations. The band observed at 1650 cm ⁻ ¹ is attributed to zeolite-bound water molecules [63]. Vibrations near 1042 cm ⁻ ¹ arise from antisymmetric Si-O-Si stretching modes associated with the zeolite’s aluminum content, with their intensity correlating to the silica-alumina ratio [64]. The peak at 789 cm ⁻ ¹ likely originates from quartz impurities or amorphous SiO₂, reflecting Si-O stretching vibrations [65]. The feature around 469 cm ⁻ ¹ result from Mg-O stretching modes. Notably, peak intensities at these three positions (1042, 789, and 469 cm ⁻ ¹) exhibit decreased magnitudes in NZ-Mg, suggesting Si-O bond cleavage and framework desilication processes.Compared to the pristine NZ zeolite, the Mg² ⁺ -modified NZ-Mg material exhibited two new peaks at 3700 cm ⁻ ¹ and 1650 cm ⁻ ¹. These bands are attributed to the vibrational modes of magnesium hydroxide (Mg(OH)₂) and the corresponding bending vibration of the O-H bond, respectively [66]. In the spectral region between 600 cm ⁻ ¹ and 800 cm ⁻ ¹, peaks typically associated with exchangeable cations were observed. However, intensity variations in this region were noted for NZ-Mg, with a reduction or even disappearance of these peaks. This phenomenon is likely due to structural modifications induced by the Mg² ⁺ modification process, including dealumination, desilication, and a decrease in crystallinity [67].

The FTIR spectra of corncob biochar (CCBC) and natural zeolite (NZ) before and after N and P adsorption are presented in Fig 9. New peaks attributable to the group vibrations of PO₄³⁻ and NH₄ ⁺ emerged at 1004 cm ⁻ ¹ and 1453 cm ⁻ ¹ for CCBC-Mg-P and NZ-Mg-P, respectively [68–70]. The absorption band at 1042 cm^-1^ is attributed to the typical asymmetric stretching vibration generated by the PO43- group and the stronger wide absorption peak resulting from the superposition of the skeleton M-O (where M = Si, Al) and the absorbed PO43- group.The peak at 876 can be assigned to the P-O-Mg complex with the A1 symmetry structure [71]. These peaks confirm the presence of N and P elements in the modified materials (CCBC-Mg-P and NZ-Mg-P). The FTIR spectra of CCBC-Mg-P and NZ-Mg-P exhibited decreased peak intensities at 445 cm ⁻ ¹ (attributed to Mg–O stretching vibrations) and 3700 cm ⁻ ¹ (attributed to Mg–OH stretching vibrations). This reduction suggests that surface Mg species were consumed during the N and P adsorption reactions [72]. The results were consistent with those obtained from scanning electron microscopy coupled with SEM and EDS analyses, which confirmed the formation of struvite (MgNH₄PO₄·6H₂O) precipitation on the material surfaces [20]. However, the FTIR spectra of CCBC-P and NZ-Mg-P revealed no new peaks corresponding to vibrational modes of PO₄³⁻ or NH₄ ⁺ , suggesting negligible N and P adsorption in CCBC-P and NZ-P. This finding underscores the critical role of Mg in facilitating PO₄³⁻ and NH₄ ⁺ adsorption.

**Fig 10:**
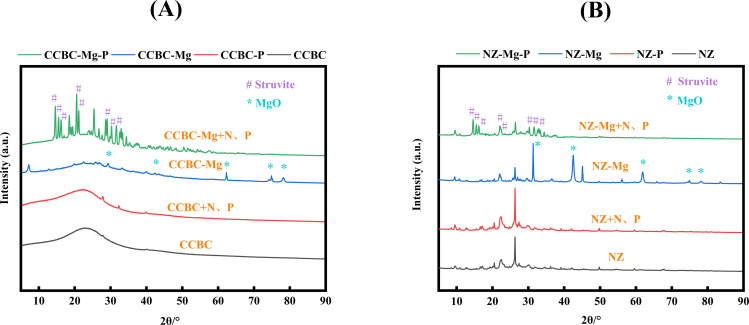
XRD images. (A): Corncob biochar-based materials. (B): Natural zeolite-based materials.

XRD analyses As shown in Fig 10, compared with the unmodified materials CCBC and NZ, the modified magnesium-containing materials CCBC-Mg and NZ-Mg can show new crystal spectral peaks at 36.9°(111), 42.9°(200), 62.3°(220), 74.7°(311), and 78.6°(222) after modification. These characteristic peaks are consistent with the MgO structure (JCPDS 45–0946) [5].

Post-domestic wastewater treatment, the crystalline phases of pristine CCBC and NZ exhibited no significant alterations. However, the Mg-modified biochars (CCBC-Mg and NZ-Mg) showed emergence of distinct spectral peaks at 2θ values of 15.04°(110), 15.85°(020), 16.50°(011), 20.91°(111), 21.50°(021), 30.66°(211), 31.97°(040), and 33.29°(022). These diffraction patterns matched precisely with the crystalline structure of struvite (MgNH₄PO₄·6H₂O) as per JCPDS card 15–762 [20]. This spectroscopic evidence confirms formation of struvite crystals on the surfaces of CCBC-Mg and NZ-Mg during wastewater remediation processes.

### Comparison with other adsorbent materials

As shown in Table 6 and Table 7, compared with the adsorption studies of AN and TP by other materials, the adsorption effects of CCBC-Mg and BZ-Mg on nitrogen and phosphorus have certain advantages. The cost of the two materials is low, the difficulty of obtaining raw materials is small, and they have certain economic benefits. In addition, the final use of struvite is farmland utilization, CCBC-Mg and NZ-Mg as struvite carrier materials are environmentally friendly and economical. CCBC-Mg and NZ-Mg can be directly used as soil amendments or fertilizers rich in nitrogen and phosphorus after absorbing nitrogen and phosphorus nutrients in water, which has certain environmental benefits. Therefore, CCBC-Mg and NZ-Mg have good application potential in the recovery of nitrogen and phosphorus nutrients in aquaculture wastewater.

**Table 6:** Comparison of CCBC-Mg with other modified biochars.

Ingredients	Types of nutrient recovered	modifiers	Adsorption capacity(mg/g)	Refernce
Bamboo	AN	FeSO_4_·7H_2_O	16.60	[73]
Pine sawdust		MgCl_2_	15.68	[74]
Sargassum		MgCl_2_	22.8 ~ 28.2	[75]
Corncob		MgCl_2_·6H_2_O	38.66	This paper.
Lychee twig	TP	FeCl_3_ ∙ 6H_2_O	19.66	[76]
Corncob		FeCl_3_ ∙ 6H_2_O and MgCl_2_ ∙ 6H_2_O	107.97	[77]
Wheat straw		Quaternary chitosan and La(NO_3_)_3_·6H_2_O	109 ± 4	[78]
Corncob		MgCl_2_·6H_2_O	116.99	This paper.

**Table 7:** Comparison of NZ-Mg with other modified biochars.

Ingredients	Types of nutrient recovered	modifiers	Adsorption capacity(mg/g)	Refernce
Zeolite	AN	FeCl_3_·6H_2_O and CH_3_COONa	3.47	[79]
Zeolite		LaCl_3_	21.5	[80]
Zeolite		NaCl	11.0 ~ 17.3	[81]
Natural zeolite		MgCl_2_·6H_2_O and NaOH	47.50	This paper.
Zeolite	TP	FeCl_3_·6H_2_O and CH3COONa	38.91	[79]
Zeolite		LaCl_3_	9.10	[80]
Natural Clinoptilolite		NaCl and LaCl_3_	1.732	[82]
Natural zeolite		MgCl_2_·6H_2_O and NaOH	103.11	This paper.

For practical applications, the raw materials used for batch production of the two materials are chemical-grade materials. The composition and sources of the materials are shown in Table 8, and the manufacturing costs of the materials are shown in Table 9. The batch manufacturing costs of CCBC-Mg and NZ-Mg are significantly lower than the commercial gypsum price of 3,300 CNY/ton, indicating that these materials have certain potential in the treatment of aquaculture wastewater. The total costs of the materials are 1,098.91 CNY/ton and 2,278.91 yuan per ton, respectively, which are lower than the Commercial struvite price of 3,300 CNY/ton, suggesting that these materials have certain potential in the treatment of livestock wastewater.

**Table 8:** Composition and Source of Materials.

Type of materials	Material composition	Quality ratio	Price (CNY/ton)	Source
CCBC-Mg	Corn cob	1	0	The experimental field has been acquired
	MgCl_2_·6H_2_O	2.033	500	Weifang Houcheng Chemical Co., Ltd. (http://www.wfhchg.cn)
NZ-Mg	Natural zeolite	1	350	Shijiazhuang Hualang Mineral Products Trading Co., Ltd. (www.zhongjingshifen.com)
	MgCl_2_·6H_2_O	2.033	500	Weifang Houcheng Chemical Co., Ltd. (http://www.wfhchg.cn)
	NaOH (32% caustic soda solution)	0.8	950	Langfang Tuodi Chemical Co., Ltd. (www.lftuodi.cn)

**Table 9:** Material Manufacturing Costs.

Type of materials	The weight loss rate after material pyrolysis	Source	Material cost (CNY/ton)	Water fee (CNY/ton)	Electricity cost (CNY/ton)	Total material cost (CNY/ton)
CCBC-Mg	Corn cob account for approximately 70%.	[83–85]	1016.5	62.1 (The industrial water cost in Beijing is 6.21 yuan per ton, and approximately 10 tons of water are needed.)	20.31 (Industrial electricity cost in Beijing is 0.6770 yuan per kilowatt-hour, and approximately 30 kilowatt-hours are required)	1098.91
NZ-Mg	Natural zeolite accounts for approximately 20%.	[86]	2196.5			2278.91

### Mechanistic for AN and TP adsorption

The adsorption mechanisms of unmodified materials (CCBC and NZ) toward ammonium nitrogen (AN) and total phosphorus (TP) in livestock wastewater involve distinct yet complementary processes. Firstly, AN adsorption occurs predominantly through ion exchange mechanisms. For CCBC, its surface contains abundant acidic functional groups (e.g., hydroxyl [-OH] and carboxyl [-COOH]), which release protons (H⁺) to facilitate ion exchange with NH₄⁺ in solution. Conversely, the NZ material exhibits weak binding affinity for alkali metal/alkaline earth metal cations (e.g., Ca² ⁺ , K ⁺ , Na⁺) on its surface, enabling efficient ion exchange with solution NH₄ ⁺ .Secondly, Regarding TP adsorption, electrostatic attraction plays a pivotal role under low-pH conditions. When the pH of the solution is between 6 and 11, the main forms of TP existing in the solution under this condition are H_2_PO^4-^ and HPO_4_^2-^ [87]. The CCBC surface undergoes protonation of its acidic functional groups (e.g., -OH and -COOH), forming positively charged moieties (-OH^2+^and -^COOH2+^) that electrostatically interact with these phosphate anions. Meanwhile, the NZ surface, enriched with cations like Ca² ⁺ , K ⁺ , and Na ⁺ , directly attracts H₂PO₄⁻ and HPO₄²⁻ through Coulombic forces.Kinetic analyses reveal that both AN and TP adsorption processes by CCBC and NZ conform closely to the pseudo-second-order model (R² > 0.99), indicating chemical adsorption as the rate-limiting step. This finding aligns with proposed mechanisms involving ion exchange, electrostatic attraction, and π-π interactions [88]. Collectively, these results confirm that unmodified CCBC and NZ materials primarily remove AN and TP from aquaculture wastewater through ion exchange and electrostatic attraction processes.

For Mg^2+^ -modified materials (CCBC-Mg and NZ-Mg), the adsorption mechanisms for ammonium nitrogen (AN) and total phosphorus (TP) in livestock wastewater involve multiple coordinated processes. Firstly, AN removal occurs predominantly through ion exchange, where surface-bound Mg^2+^ on both CCBC-Mg and NZ-Mg readily exchange with solution NH^4 +^ . This mechanism is facilitated by the high availability of exchangeable Mg^2 +^ sites on the modified materials.Secondly, TP adsorption proceeds via electrostatic attraction.Upon contact with aqueous solution, surface Mg species on CCBC-Mg and NZ-Mg undergo in-situ hydrolysis at the solid-liquid interface, generating additional Mg^2 +^ . The resulting positively charged material surfaces effectively attract the predominant TP species in solution (H2PO_4_^-^ and HPO_4_^2-^) under circumneutral pH conditions (6–11), forming electrostatic complexes.Notably, CCBC-Mg and NZ-Mg achieve simultaneous AN and TP recovery through struvite (MgNH4PO4·6H2O) precipitation.The Mg^2 +^ released from surface Mg compounds reacts with residual NH_4_^+^ and PO_4_^3-^ in wastewater, forming struvite crystals that deposit on the material surfaces. While ion exchange and electrostatic attraction provide limited recovery capacity [89,90], struvite precipitation constitutes the primary recovery mechanism, underscoring the critical role of Mg modification in enhancing simultaneous AN and TP removal efficiency for corn cob biochar (CCBC) and natural zeolite (NZ) materials.

### Evaluate biochars and natural zeolite as potential soil conditioner

Previous studies have demonstrated that integrating biochar with inorganic or organic fertilizers in crop production systems significantly enhances crop yields. This phenomenon is attributed to the capacity of biochar to adsorb ammonium ions from conventional fertilizers, enabling their gradual release into the soil matrix and thereby improving plant uptake efficiency [91]. Furthermore, biochar application mitigates nutrient leaching losses (notably ammonium, potassium, and phosphorus ions), as adsorbed ammonium within biochar structures exhibits superior utilization rates compared to traditional ammonium-based fertilizers. The strong affinity of biochar for NH₄⁺ and its exceptional nutrient retention capacity suggest that waste-derived biochar holds considerable promise as an eco-friendly technology for enhancing soil physicochemical properties and agricultural productivity [92]. It should be noted, however, that optimal biochar application rates remain context-dependent, varying according to soil type and crop species.

The utilization of nutrient-enriched (eutrophic) biochar in agricultural systems offers dual benefits: reducing external fertilizer inputs while serving as an effective slow-release nutrient source for plants. Empirical evidence indicates that such biochar formulations enhance plant disease resistance, germination rates, and nutrient absorption efficiency, while concurrently minimizing nitrogen leaching [28,29,31,93]. Nutrient-rich biochar materials undergo post-adsorption mineralization processes, forming struvite (MgNH₄PO₄·6H₂O) crystals that remain physically entrapped within the biochar matrix [94]. This characteristic enables phosphorus fertilization without risk of secondary environmental contamination. When eutrophic biochar is incorporated into soil systems, it modulates nitrogen and phosphorus dynamics through multiple mechanisms: pH adjustment, physical adsorption of N/P species, accelerated mineralization processes, and enhanced cation exchange capacity [95,96]. Consequently, soil analyses following eutrophic biochar application reveal significant increases in available nitrogen and phosphorus concentrations, suggesting that biochar modifies soil-crop nutrient cycling pathways to favor plant uptake [97,98]. These findings collectively demonstrate that nitrogen- and phosphorus-loaded eutrophic biochar functions as a plant growth promoter with potential applications as a carbon-based fertilizer in agricultural settings.Nevertheless, further investigation is required to fully characterize the release kinetics of nitrogen and phosphorus from eutrophic biochar under field conditions.

Previous research has established that eutrophic zeolites demonstrate significant potential for enhancing soil physicochemical properties, optimizing nutrient utilization efficiency, and mitigating carbon-nitrogen (C-N) gas losses from agricultural systems [99,34]. However, contrary to conventional slow-release fertilizers, the nitrogen present in eutrophic zeolites exhibits immediate bioavailability to soil microorganisms. This rapid accessibility can stimulate soil nitrification processes, necessitating the co-application of nitrification inhibitors when utilizing eutrophic zeolites as soil amendments or fertilizers.

The utilization of zeolites as soil conditioners underscores their beneficial modulation of soil nitrogen dynamics. Empirical evidence suggests this intervention may enable reductions in nitrogenous fertilizer inputs while simultaneously minimizing nitrate (NO_3_^-^) leaching losses-outcomes that could maintain or even enhance crop yields depending on agronomic context [32,100]. To fully realize this potential, expanded experimental validation is required across: (1) diverse zeolite varieties (e.g., clinoptilolite), (2) contrasting soil textures (notably sandy soils), (3) extended cultivation durations, and (4) critical environmental variables including temperature regimes, moisture dynamics, and crop presence. Future investigations should prioritize elucidating the long-term impacts of natural and eutrophic zeolites on soil total nitrogen regimes under field conditions.

Furthermore, both natural and nutrient-enriched zeolites enhance soil cation exchange capacity (CEC) while inducing transient reductions in microbial carbon (C) and nitrogen (N) pools. This microbial perturbation appears to be followed by potential fungal population expansion, though direct plant uptake of zeolite-derived nitrogen remains unlikely. The complex interplay governing nutrient transfer likely involves intricate soil-microbe-plant interactions, warranting dedicated investigation into these biological mediation mechanisms [101].

## Future work

Firstly, the scope of this investigation was restricted to nitrogen and phosphorus recovery from simulated livestock wastewater. Future research should extend to biogas slurry systems to validate these findings under real-world conditions.Secondly, regarding adsorption mechanism characterization, the experimental analysis was limited to quasi-first-order (QFO) and quasi-second-order (QSO) kinetic models, alongside Langmuir and Freundlich isothermal adsorption frameworks.A comprehensive examination of alternative kinetic and thermodynamic models is warranted to establish a more robust mechanistic understanding of the synchronous recovery processes for nitrogen and phosphorus in livestock wastewater systems using CCBC-Mg and NZ-Mg materials.Finally, the nutrient release dynamics of magnesium-modified biochar fertilizers (CCBC-Mg-P and NZ-Mg-P) in soil environments require systematic investigation. This constitutes a critical evaluation parameter for assessing their viability as sustainable green fertilizers in agricultural applications.

## Conclusions

Two distinct matrix materials (corn cob and natural zeolite) underwent Mg² ⁺ -modification to synthesize CCBC-Mg and NZ-Mg composite materials respectively. Unmodified counterparts (CCBC and NZ) without magnesium supplementation served as control groups, enabling comparative analysis of the impact induced by Mg² ⁺ modification on their nutritional recovery capabilities.A one-way ANOVA was systematically employed to compare the effectiveness of each material in simultaneously recovering N and P from LPFE. The results obtained demonstrated that Mg^2 +^ modification significantly enhanced the simultaneous recovery of N and P by CCBC-Mg and NZ-Mg. CCBC-Mg was found to be more suitable for the recovery of P from LPFE, while NZ-Mg was more effective for the recovery of AN.Characterizations via BET, SEM, FTIR analyses and XRD analyses revealed distinct magnesium (Mg) loading configurations on the surfaces of CCBC-Mg and NZ-Mg materials.Notably, the corresponding precipitation products exhibited struvite crystal formation on their surfaces. To elucidate the adsorption mechanisms of AN and TP by these materials, adsorption kinetics and isothermal modeling were employed. The findings demonstrated that the adsorption processes for both AN and phosphate across all materials adhered to the pseudo-second-order kinetic model and Langmuir isotherm, collectively indicating that monolayer adsorption governed by chemical interactions was the predominant mechanism.Maximum adsorption capacities for AN followed the order: NZ-Mg (47.50 mg/g)> CCBC-Mg (38.66 mg/g)> NZ (10.72 mg/g)> CCBC (7.15 mg/g). For TP, the hierarchy was: CCBC-Mg (116.99 mg/g)> NZ-Mg (103.11 mg/g)> CCBC (8.45 mg/g)> NZ (5.38 mg/g). These findings highlight the material-specific response to Mg² ⁺ modification and inherent adsorptive preferences.Mechanistic investigations revealed that CCBC-Mg and NZ-Mg utilized multiple adsorption mechanisms, with struvite precipitation being dominant, accompanied by physical adsorption, ion exchange, and electrostatic attraction. Mg² ⁺ modification elevated solution pH and promoted Mg-P precipitate formation on material surfaces, facilitating favorable alkaline conditions and nucleation sites for struvite crystallization. This alkaline microenvironment significantly enhanced concurrent N and P recovery. In contrast, unmodified CCBC and NZ primarily relied on electrostatic interactions and ion exchange processes for AN and TP adsorption.

## Supporting information

S1 Fig Nitrogen and phosphorus adsorption properties of natural zeolite materials modified with different Mg^2 +^ concentrations at 400°C. (TIF)

S1 Appendix The minimum dataset of this paper. (ZIP)
