# Supplementary figures and images for: Mg²⁺-modified corncob biochar and natural zeolite significantly enhanced simultaneous N and P recovery from livestock wastewater
Source: PLoS One. 2025 Aug 29;20(8):e0331575. doi: 10.1371/journal.pone.0331575 (PMC12396728; doi:10.1371/journal.pone.0331575)

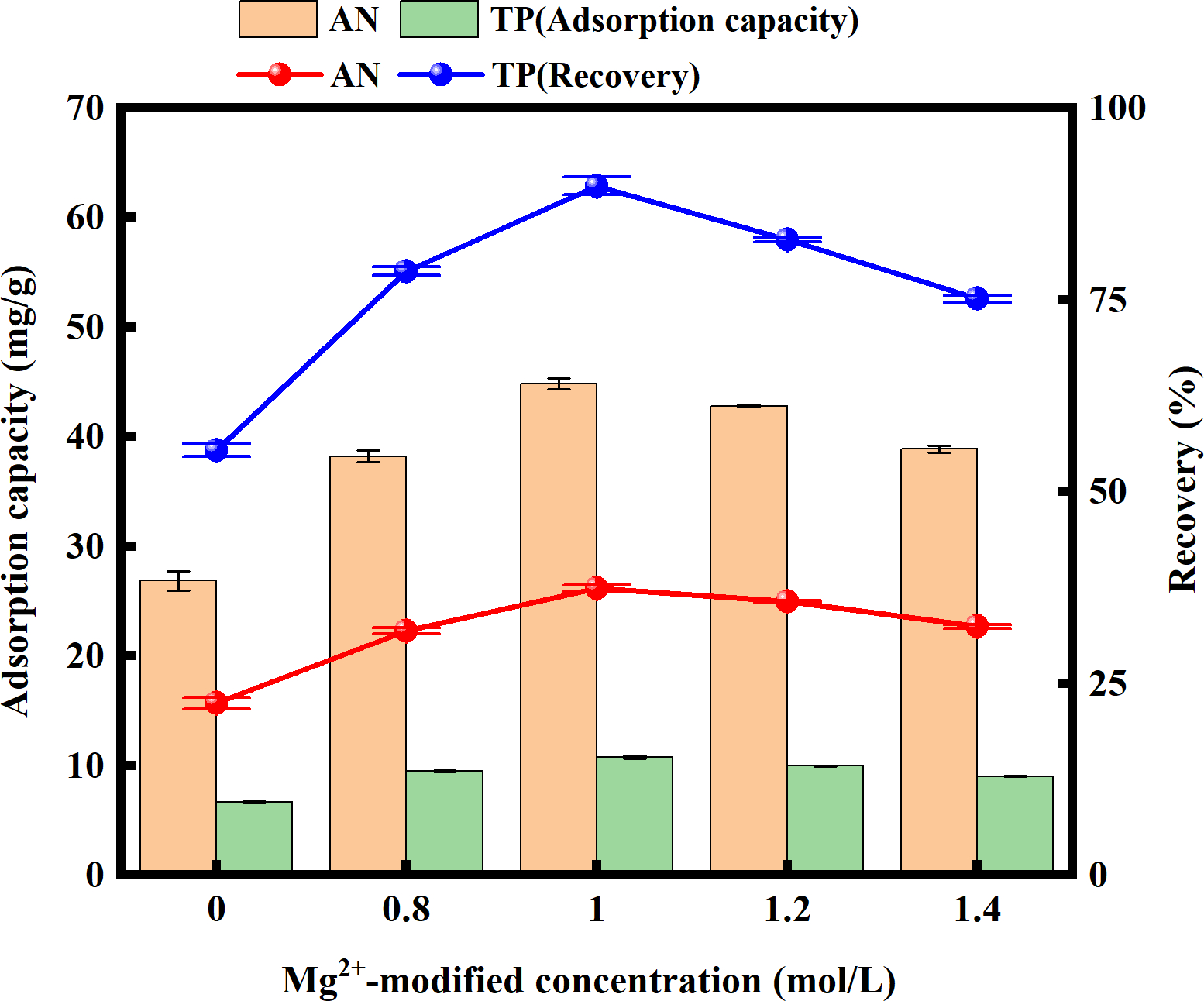

Supplement: S1 Fig — (TIF) [file pone.0331575.s001.tif]
